# Tapping to a Slow Tempo in the Presence of Simple and Complex Meters Reveals Experience-Specific Biases for Processing Music

**DOI:** 10.1371/journal.pone.0102962

**Published:** 2014-07-30

**Authors:** Sangeeta Ullal-Gupta, Erin E. Hannon, Joel S. Snyder

**Affiliations:** Department of Psychology, University of Nevada Las Vegas, Las Vegas, Nevada, United States of America; National University of Singapore, Singapore

## Abstract

Musical meters vary considerably across cultures, yet relatively little is known about how culture-specific experience influences metrical processing. In Experiment 1, we compared American and Indian listeners' synchronous tapping to slow sequences. Inter-tone intervals contained silence or to-be-ignored rhythms that were designed to induce a simple meter (familiar to Americans and Indians) or a complex meter (familiar only to Indians). A subset of trials contained an abrupt switch from one rhythm to another to assess the disruptive effects of contradicting the initially implied meter. In the unfilled condition, both groups tapped earlier than the target and showed large tap-tone asynchronies (measured in relative phase). When inter-tone intervals were filled with simple-meter rhythms, American listeners tapped later than targets, but their asynchronies were smaller and declined more rapidly. Likewise, asynchronies rose sharply following a switch away from simple-meter but not from complex-meter rhythm. By contrast, Indian listeners performed similarly across all rhythm types, with asynchronies rapidly declining over the course of complex- and simple-meter trials. For these listeners, a switch from either simple *or* complex meter increased asynchronies. Experiment 2 tested American listeners but doubled the duration of the synchronization phase prior to (and after) the switch. Here, compared with simple meters, complex-meter rhythms elicited larger asynchronies that declined at a slower rate, however, asynchronies increased after the switch for all conditions. Our results provide evidence that ease of meter processing depends to a great extent on the amount of experience with specific meters.

## Introduction

We are continually confronted with sensory input that must be interpreted before meaningful interaction with the world can occur. People adapt flexibly to environmental contexts by drawing on prior experiences to efficiently encode and respond appropriately to novel experiences or stimuli. Specific experiences that vary across cultures may lead individuals to perceive and respond to novel stimuli in culture-specific ways [1], [2]. Moreover, processing may become optimized for culturally familiar structures because of long-term exposure. Language provides an obvious example of this: from as early as one year of age, we are better at discriminating and producing speech sounds from our native language than sounds from a foreign language [3], [4]. Similarly, we more readily discriminate faces of our own race or species than other-race or non-human-primate faces [5]–[7]. In music, it is easier to detect mistuned notes within the context of a familiar than foreign musical scale [8], [9]. The present work provides novel evidence that everyday musical behaviors such as listening and dancing to music in a specific cultural context (specifically, India versus the United States) can give rise to robust cross-cultural differences in perception and production of temporal information among adult listeners.

Tapping and dancing are ubiquitous human musical activities, yet relatively little is known about the nature of the cognitive mechanisms that enable such behaviors [10]–[12]. To synchronize movements to music, a listener must perceive its *meter*, which is subjectively experienced as an underlying pattern of strong and weak beats [13]. For example, a waltz has a repeating cycle of three beats (***ONE*** *-two-three,* ***ONE*** *-two-three*) whereas a march has a repeating cycle of four beats in groups of two (***ONE*** *-two-* ***three*** *-four*). Because meters vary across cultures, the ease with which listeners perceive and produce rhythmic patterns may depend on their culture-specific exposure to different meters. Western “simple” musical meters are dominated by an even, or isochronous, beat that can be subdivided or multiplied by simple integers to produce other levels of a metrical hierarchy. Because rhythmic events tend to occur on metrically strong positions, rhythms that conform to simple meters tend to have long and short temporal intervals related by simple-integer ratios such as 2∶1 or 3∶1. Western listeners more accurately perceive and produce rhythmic patterns containing simple ratios than those containing complex ratios such as 3∶2 or 7∶4 [14]–[19].

Non-isochronous, “complex” meters, by contrast, are dominated by a non-isochronous beat of alternating long and short durations, and rhythms that conform to complex meters are more likely to contain integer ratios such as 3∶2. These complex meters are common in music throughout South Asia, Africa, the Middle East, and Eastern Europe [20]–[21]. In Indian classical music, for instance, although the most commonly occurring rhythmic pattern is *Teental*, an isochronous duple meter pattern, other non-isochronous patterns also occur frequently, such as *Jhaptal*, which is comprised of alternating units of 3 and 2 [22]. This observation raises the natural question of whether listeners from these cultures, who have greater exposure to non-isochronous complex meters, would also exhibit processing advantages for rhythms that conform to isochronous simple meters.

To date, only a few studies have investigated rhythm processing among listeners from non-Western cultures. Unlike Western listeners, who fail to detect disruptions in sequences with a complex meter, adults from Macedonia and Bulgaria readily detect disruptions in sequences conforming to either simple or complex meters [23], [24]. This indicates that Western listeners have difficulty perceiving rhythmic patterns with complex-integer ratios primarily because they are inconsistent with familiar, simple meters. On the other hand, even listeners who are familiar with complex meters have difficulty processing sequences composed of highly complex integer ratios such as 7∶4 [25]. Interestingly, even infant listeners, who tend to exhibit less cultural bias, have difficulty with highly complex rhythms [26], as do trained musicians [14], suggesting that non-isochronous meters and complex-ratio rhythms might be categorically different from other meters [27] and intrinsically challenging to the human perceptual system.

If simple meters are easier to infer and maintain, they should lead to more accurate and faster rates of synchronization, compared to complex meters. Further, the familiar simple meters should be more resistant to reorganization than unfamiliar complex meters. Immediately upon presentation of a rhythmic pattern, listeners usually begin to interpret events according to a metrical framework [13]. Once a meter is firmly established in the mind of the listener, novel rhythmic events that do not conform to the established meter give rise to syncopation, or the feeling that events are “off-beat” until such events force a cognitive shift, such as inferring an alternative metrical framework [13], [27]. Rhythms with the highest amounts of syncopation are more difficult to reproduce, even by trained musicians [28]. Given that syncopation is typically defined by the degree to which accented events do or do not correspond to the location of the inferred metrical pulse, syncopation can only occur when the listener has inferred a meter. Syncopation might thus be considered a hallmark of metrical processing [13], [27], [29]. Effects of syncopation on perception and production should therefore be strongest when the listener has inferred a robust metrical framework. Accordingly, if complex meters are intrinsically and universally challenging, then we might expect listeners to fail to activate any metrical framework or activate only a weak framework, and syncopation should therefore minimally impact perception and production, regardless of the listeners' cultural background. By contrast, if long-term culture-specific exposure to both simple and complex meters allows non-Western listeners to readily infer either type of metrical framework, syncopation should similarly destabilize performance among such listeners when either a simple or a complex metrical framework has been inferred.

The present study employs a novel task design that demonstrates the stabilizing effects of simple and complex meters, and the effects of switching from one meter to another following stabilization. We examine sensorimotor synchronization (i.e., tapping in synchrony with a regular pattern), which is a sensitive, on-line measure of rhythm processing that could potentially uncover simple-meter biases not previously observed in other studies comparing Western and non-Western listeners, which used coarser measures of musical rhythm perception cf. [23], [24], [26].

Participants were asked to tap in synchrony with target events occurring every 3 s, a task we expected would be challenging because such slow sequences are typically difficult to perceive and produce precisely [30]–[32]. On some trials, inter-target intervals were filled with silence. However, on most trials inter-target intervals were filled with to-be-ignored simple- or complex-meter drum patterns. Because drum patterns subdivide intervals into more manageable segments [33]–[37], we expected listeners to tap more synchronously in the presence of drum patterns, despite instructions to ignore them. We expected greater synchrony and faster rates of decline during tapping to drum patterns that are consistent with a familiar metrical framework. An additional subset of filled trials examined the disruptive effects of suddenly switching the subdividing drum pattern from one meter to another. If the initial drum pattern activates a robust sense of meter, a sudden switch to a new pattern should sound syncopated and destabilize synchronization performance. The incurred cost of switching should therefore indirectly reflect the strength of the inferred meter activated prior to the switch. Our design therefore allows us to probe the strength of culture-specific metrical processing by highlighting how such processing enhances synchronization *and* how contradictory rhythmic information disrupts synchronization.

## Materials and Methods

### Ethics Statement

All procedures were approved by UNLV's Institutional Review Board for Human Subjects Research (Social/Behavioral), and complied with the ethical guidelines of the Office of Research Integrity. Written informed consent was obtained from all participants.

### Experiment 1

#### Participants

American participants were college students from Las Vegas, Nevada, USA (*N* = 51, *M* = 23.2 years, 14 male, 35 female, 2 undisclosed) who participated for course credit. Their music training ranged from 0–7 years (*M* = 3.4, *SD* = 2.62), with 15 participants reporting 0 years of music training. Indian college students were recruited from Bangalore, India (*N* = 51, *M* = 22.6 years, 18 male, 33 female) with 0 to 15 years of music training (*M* = 4.7, *SD* = 4.26) with 11 participants reporting 0 years of music training. All participants reported normal hearing.

#### Apparatus and Stimuli

Unfilled baseline sequences consisted of 11 sine tones at 500-Hz and 100-ms in duration (0-ms rise and fall time), which were repeated every 3 s yielding a 3-s inter-onset interval (IOI). The 2900-ms interval between tones was silent.

In filled baseline sequences, the 2900-ms interval between tones contained 12 *tabla* (Indian drum) beats having a 250-ms IOI. Drum patterns had no offset-to-onset interval (aside from the natural decay of intensity in the timbre), to make the sounds as musical as possible. Weak beats used the *khali* timbre and strong beats used the *tali* timbre accented by doubling the amplitude. Sequences were created using the music software, Swarsystems, and converted to AIFF using Audacity, a digital audio editor.

**Figure 1:**
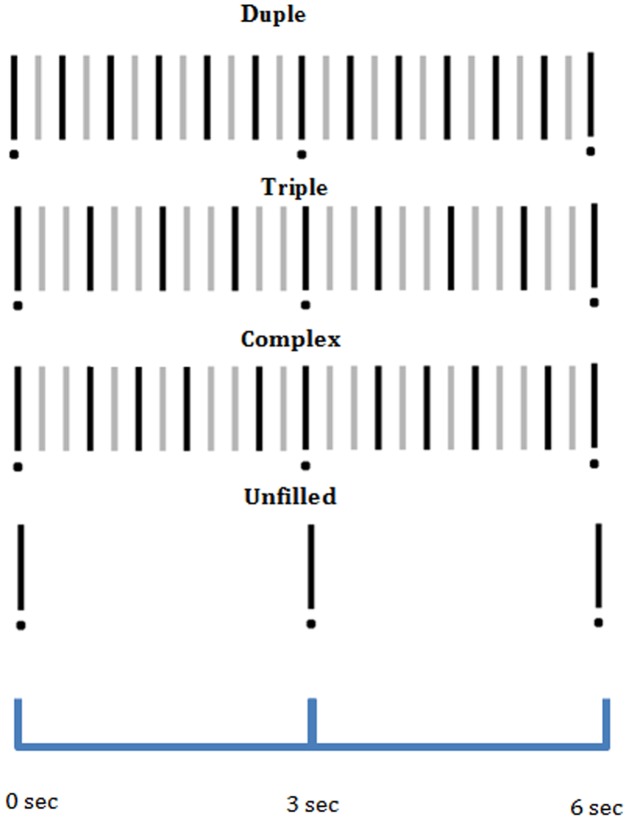
Schematic diagrams of two cycles of the three patterns (each cycle repeated ten times in experiment). Dots represent the target tap location for synchronization. Vertical bars represent drum beats, with strong beats indicated in black and weak beats indicated in gray.

There were three metrical arrangements of strong and weak beats that could be imposed on the isochronous drum sounds. The simple duple pattern subdivided the inter-target interval into six groups of two by alternating between one strong and one weak beat (2+2+2+2+2+2). The simple triple pattern subdivided the inter-target interval into four groups of three by alternating between one strong and two weak beats (3+3+3+3). The third, complex pattern subdivided the inter-target interval into a pattern of groups of two and three beats (3+2+2+3+2). Figure 1 provides a schematic diagram of the three drum patterns. Simple duple and triple patterns were expected to be equally familiar to American and Indian listeners, whereas the complex pattern was only expected to be familiar to Indian listeners [20].

**Figure 2:**
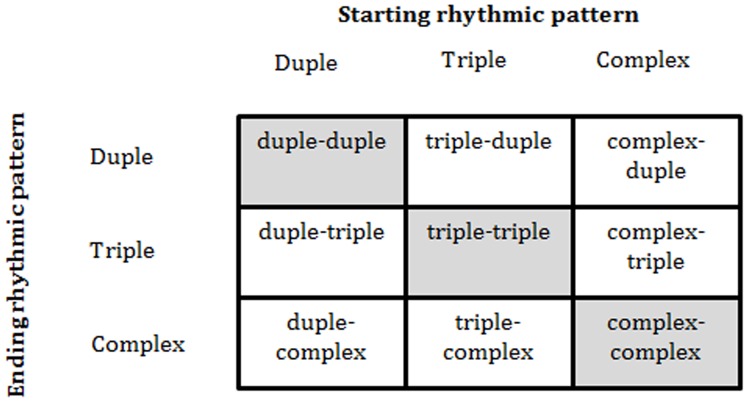
Schematic diagram of filled baseline and switch patterns. “Starting drum pattern” corresponds to the rhythmic structure of the first five cycles; “Ending drum pattern” corresponds to the rhythmic structure for the last five cycles. Shaded boxes indicate sequences in which the starting rhythmic pattern is the same as the ending rhythmic pattern (filled baseline).

All sequences were 30 s in duration (10 cycles). For filled baseline sequences, the drum pattern remained constant over the 10 cycles. For filled switch sequences, one drum pattern played for five cycles and a different pattern played during the last five cycles. We assumed that if the starting drum pattern induced a robust perception of meter, the ending drum pattern should sound relatively syncopated, at least immediately after the switch and before a new metrical percept could emerge. Specifically, the patterning of accents (as demarcated by the *tali* timbre) in the ending rhythm contradicted those of the starting pattern, with unexpected events (i.e., no accent when an accent was expected or vice versa) occurring 40–60% of the time. Figure 2 describes all filled sequences.

Stimuli were presented binaurally over stereo noise-cancelling Phillips SBC HN110 headphones at a comfortable intensity by a Boss Micro-BR Roland Digital Recorder, which also simultaneously recorded taps as digital acoustic data.

#### Procedure

Participants tapped on the digital recorder near the microphone at a location marked with tape. Participants were instructed, “You will hear repeating tones like this [tone played].Your job is to tap with every tone. In between the tones, you might hear other intervening beats, but you should ignore these and simply tap along with the target tone.” Each trial began with one complete cycle before the first target tone occurred. Before the experiment, the participants completed a practice trial in which they tapped to a randomly selected filled baseline sequence. They were allowed to repeat the practice trial if they chose to, although no participant did. Following practice, the experiment progressed in three blocks. During the first and last blocks, the participant tapped to unfilled baseline sequences. The middle block consisted of 18 filled trials. Each filled baseline and filled switch sequence occurred twice. The order of presentation within each section was randomly assigned, with the constraint that the same sequence never repeated consecutively.

#### Data Analysis

Tap times were extracted from the acoustic waveform by using a custom-made onset detection algorithm in Matlab to extract the temporal positions of peak amplitudes in the digital recording of taps relative to the start of the trial. All measures were derived from circular statistics [38]. The IOI of 3 s was transformed into a circular scale, and all tap times in ms were assigned a point on the circular scale by dividing the tap time (in ms) by the IOI (3000 ms) and multiplying the quotient by 360. Thus, the relative phase is defined as the difference between the tap position and the nearest target event (in degrees) normalized for cycle period [39].

As a measure of tapping variability, we computed angular variability, as defined by the mean resultant length (MRL) which is analogous to the coefficient of variation for linear data. It is defined as follows [39], [40]:

X = ∑(^cosθ*j*^ */_n_*) Y = ∑(^sinθ*j*^ */_n_*) MRL = √(X^2^+Y^2^)

where *j* refers to tap position (in radians), and n refers to the number of taps. MRL is an inverse measure of the variability within the relative phases in a trial, such that a higher MRL corresponds to lower variability [41].

Finally, a relaxation time measure was calculated to provide an estimate of how long it takes for relative phase to decline towards the target, as the participant transitions from responding to the stimulus at the very start of the trial to synchronizing with it. Presumably relaxation time provides a sense of how readily a given metrical representation can be accessed and utilized to guide behavior. The relaxation time of relative phase values over the course of a trial can be defined by the time constant (τ) and described by an exponential decay function [42], with a larger τ corresponding to a slower decline in relative phase through the course of the trial [43]. In order to calculate τ, relative phase values (in degrees) corresponding to the first 5 taps were used to estimate the exponential function describing the curve. From the resulting equation, τ was calculated by estimating the tap position at which the relative phase value would reach a value of 1 degree.

## Results

### Baseline trials

#### Relative Phase

**Figure 3:**
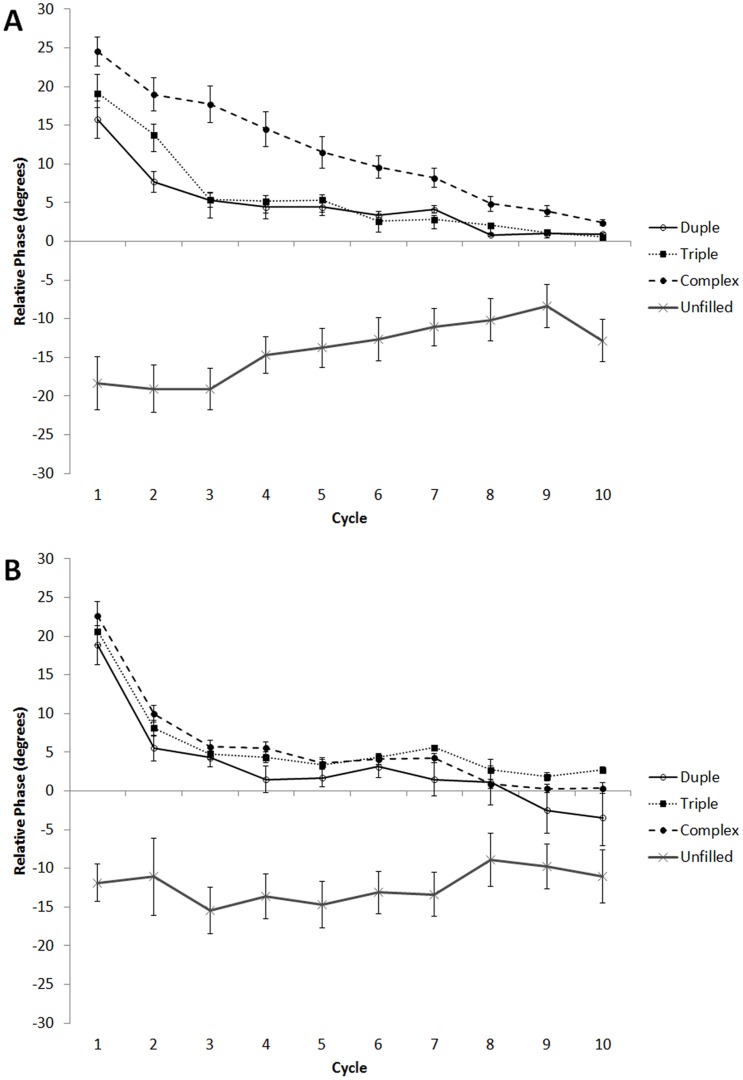
Mean average relative phase over course of trial for baseline sequences in Experiment 1 for Americans (A) and Indians (B). Error bars denote between-subject standard error. Some error bars were too small to be visible.

We excluded from analysis highly erroneous taps (0.15% of all taps) that were over five standard deviations from the mean. For each participant, relative phase values (in degrees) for each type of sequence were averaged across both repetitions. Figure 3 presents average tap-tone relative phase values for each target tap position of baseline sequences for American (Figure 3A) and Indian (Figure 3B) participants.

For both groups, relative phase remained consistently negative (preceded targets) over the course of unfilled baseline sequences. This anticipatory tendency is consistent with prior studies using long, unfilled intervals [44]. During filled baseline sequences, relative phase was initially positive (followed targets) and gradually decreased towards 0 over the course of the trial. For Indian participants, relative phase decreased similarly over the course of filled baseline trials, regardless of whether the rhythm conformed to a duple, triple, or complex meter. By contrast, for American participants, relative phase values were similar for duple and triple baseline sequences, but the complex sequence yielded higher relative phase values that took longer to decline towards 0 over the course of the trial.

**Figure 4:**
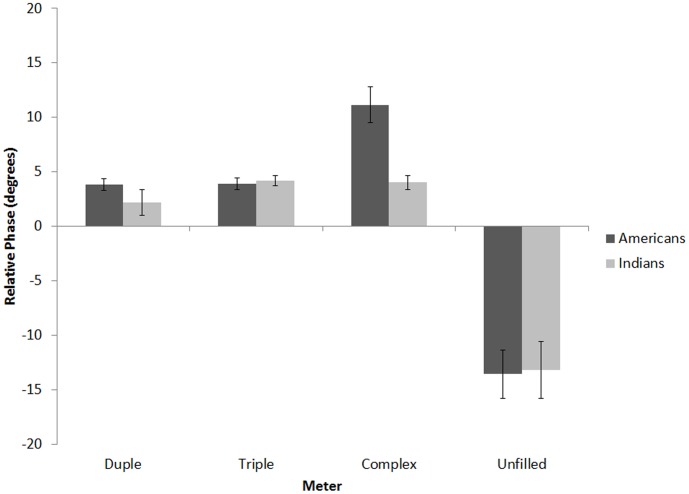
Mean relative phase for unfilled, duple, triple, and complex baseline sequences for Americans and Indians in Experiment 1. Error bars denote between-subject standard error.

For statistical analyses, we excluded the first two taps, when subjects were initially finding the beat [45], as well as the last two taps, to equalize number of data points before and after the switch. Mean relative phase over tap positions 3–8 was calculated for each sequence type (Figure 4) and submitted to a 4×2 (Sequence [unfilled, duple, triple, complex] within-subjects, x Nationality [Indian, American] between subjects) mixed-design ANOVA, with Music Training (in years) entered as a covariate. Since the Indian group had more music training than the American group, using music training as a covariate allowed us to control for its potential contribution to group effects. This analysis yielded main effects of Sequence, *F*(3,97) = 54.30, *p*<.001, *η_p_2* = .35, with unfilled trials having a significantly larger (in magnitude) relative phase (*M* = −13.40, *SD* = 17.16) than any of the filled trials (*M*<3.02, *SD*<9.67). Unfilled trials showed a negative (anticipatory) relative phase, whereas filled trials showed a positive (reactive) relative phase value. There was also a significant main effect of Music Training, *F*(1,99) = 5.09, *p*<.05, *η_p_2* = .049. Simple correlations showed that mean relative phase and years of music training were negatively correlated for duple, *r*(102) = .23, *p*<.05, triple, *r*(102) = .37, *p*<.01, and complex, *r*(102) = .37, *p*<.01 sequences, but uncorrelated for unfilled sequences, *r*(102) = .02, *p* = .83. Thus, those with more music training tended to tap closer to the targets, at least during filled trials.

To examine effects of the accompanying rhythm during filled trials, relative phase values for each of the filled sequence types were submitted to a 3×2 (Sequence [duple, triple, complex] within-subjects, x Nationality [Indian, American] between subjects) mixed-design ANOVA, with Music Training (in years) entered as a covariate. Results revealed a main effect of Sequence, *F*(2,198) = 19.37, *p*<.001, *η_p_2* = .16, and a significant interaction between Sequence and Nationality, *F*(2,198) = 11.65, *p*<.001, *η_p_2* = .11. Separate ANOVAs were then conducted for each filled sequence type with Nationality (American, Indian) as the lone between-subjects factor and Music Training as a covariate, revealing that while Indian and American participants did not differ on their relative phase values for duple, *F*(1,99) = 0.73, *p* = .39, *η_p_2* = .007, or triple sequences, *F*(1,99) = 1.54, *p* = .22, *η_p_2* = .015, for complex-meter sequences Americans had significantly higher relative phase (*M* = 11.15°, *SD* = 11.95) than Indian listeners (*M* = 4.01°, *SD* = 4.46), *F*(1,99) = 12.17, *p*<.001, *η_p_2* = .10. This suggests that when filled baseline sequences conformed to culturally familiar meters, participants exhibited greater synchrony.

#### Tapping Variability

**Figure 5:**
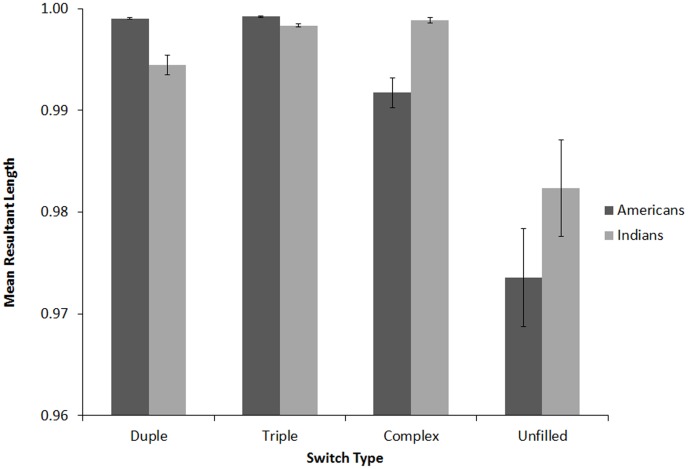
Angular variability (MRL) for unfilled, duple, triple, and complex baseline sequences for Americans and Indians in Experiment 1. Larger values signify lower variability. Error bars denote between-subject standard error.

As described above, MRL is analogous to the coefficient of variation for linear data, with larger lengths reflecting lower variability and smaller lengths reflecting higher variability of performance over the course of a given trial. We used the same window of taps to calculate MRL that we did for relative phase, omitting the first two and last two taps in each trial. Thus, taps 3–8 were used to calculate MRL in the baseline trials, and taps 3–5, and 6–8 were used to calculate the pre- and post-switch trials respectively for the switch trials. For each participant, MRL values for each condition were averaged across the two trial repetitions. As shown in Figure 5, lengths were generally close to 1, suggesting relatively low variability or highly consistent tapping for all participants in all conditions. MRL values for each trial type were submitted to a 4×2 (Sequence [unfilled, duple, triple, complex], within-subjects, x Nationality [Indian, American], between subjects) mixed-design ANOVA, with Music Training (number of years reported) as a covariate. There was a significant main effect of Sequence, *F*(3,97) = 24.15, *p*<.001, *η_p_2* = .20, and Music Training, *F*(1,99) = 7.76, *p*<.01, *η_p_2*<.073. The unfilled trials had a significant lower MRL (higher variability) (*M* = 0.97, *SD* = .035) than any of the filled trials (*M*>.995, SD<.008). Simple correlations showed that MRL and years of music training were positively correlated for unfilled, *r*(102) = .21, *p*<.05, and complex sequences, *r*(102) = .30, *p*<.01, but uncorrelated for duple, *r*(102) = .13, *p* = .19, triple, *r*(102) = .008, *p* = .93. Thus, those with more music training tended to tap with less variability, but only on the complex and unfilled sequences.

Next, to examine the effects of rhythm during filled trials, MRL for each of the filled sequence types were submitted to a 3×2 (Sequence [duple, triple, complex] within-subjects, x Nationality [Indian, American] between subjects) mixed-design ANOVA, with Music Training (in years) entered as a covariate. This yielded a significant interaction between Sequence and Nationality, *F*(1,99) = 34.22, *p*<.001, *η_p_2* = .26. ANOVAs conducted separately for each filled sequence type with Nationality (American, Indian) as a between-subjects factor and Music Training as a covariate, revealed significantly larger MRL (less variability) for the Indian participants in the complex sequences, *F*(1,102) = 19.40, *p*<.001, *η_p_2* = .16, but significantly smaller MRL (greater variability) in the duple, *F*(1,102) = 28.04, *p*<.001, *η_p_2* = .22, and triple sequence conditions, *F*(1,102) = 31.91, *p*<.001, *η_p_2* = .24. The variability findings in the complex sequence trials align with those of relative phase: compared with Indian listeners, American listeners were more variable when synchronizing with the complex sequence trials.

#### Relaxation Time

Any τ values that were more than 5 standard deviations away from the overall mean were excluded as outliers. An additional 0.23% of all the τ values were discarded for this purpose. Since baseline and switch trials are identical until the 6^th^ target event, we combined the first 5 taps across baseline and switch trials for a given starting meter. The τ values were first calculated for each participant for each trial type (using mean relative phase over the two repetitions), and these values were then averaged over the trial types based on starting sequence (duple, triple, complex), regardless of whether they were switch or baseline trials. This resulted in τ values for starting duple, starting triple, and starting complex sequences.

**Figure 6:**
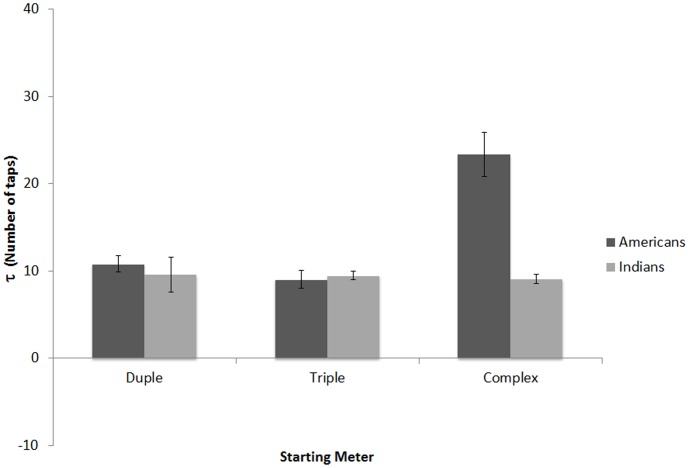
Relaxation time: τ (in number of taps) for sequences starting with duple, triple and complex sequences for Americans and Indians in Experiment 1, averaged over baseline and switch trials. Values represent an estimate of the number of cycles (taps) required for the relative phase to reach 1 degree. Error bars denote between-subject standard error.

As seen in Figure 6 (also see Figure 3), Indian participants had relatively low τ values over the three sequence types. In contrast, American participants had relatively low τ values across duple and triple sequence conditions, but higher τ values for the complex sequence condition. This suggests that Indian participants showed a similar rate of decline in relative phase across all three conditions, whereas American participants showed a slower rate of decline in complex sequence trials compared to either of the simple sequence trials (i.e. it took longer for tapping to stabilize).

To examine these trends, τ values were submitted to a 3×2 (Starting Sequence [duple, triple, complex] within-subjects, x Nationality [Indian, American] between subjects) mixed-design ANOVA, with Music Training (in years) entered as a covariate. There were significant main effects of Nationality, *F*(1,95) = 14.36, *p*<.01, *η_p_2* = .13, Starting Sequence, *F*(2,190) = 20.65, *p*<.001, *η_p_2* = .18, and Music Training, *F*(1,95) = 10.58, *p*<.01, *η_p_2* = .10. The effects for Nationality were driven by Americans having an overall larger τ value (M = 14.27, SD = 13.00) than Indians (M = 9.37, SD = 8.72). Bonferroni corrected paired-sample t-tests revealed differences between complex (M = 15.92, SD = 14.25) and duple (M = 10.17, SD = 11.10) sequences, *t*(97) = 2.96, *p*<.01, and between complex and triple (M = 9.25, SD = 5.69) sequence, *t*(97) = 5.15, *p*<.001, but no difference between duple and triple sequence conditions, *t*(99) = .70, *p* = .48. Music training negatively correlated with τ values for the complex, *r*(98) = −.41, *p*<.001, and triple, *r*(100) = −.35, *p*<.001, conditions, but not for the duple condition, *r*(100) = −.31, *p* = .10, *r*(100) = −.17, *p* = .093, suggesting that with music training, listeners required a shorter relaxation time, at least for the complex and triple sequences.

There was also an interaction between Starting Sequence and Nationality, *F*(2,190) = 14.74, *p*<.001, *η_p_2* = .13. Subsequent ANOVAs conducted separately for each sequence type with Nationality (American, Indian) and Music Training as a covariate, revealed that while Indian and American participants did not differ when the trial started with duple, *F*(1,97) = .52, *p* = .47, *η_p_2* = .005, or triple sequences, *F*(1,97) = 1.11, *p* = .29, *η_p_2* = .001, Americans had significantly higher τ (*M* = 23.37 taps, *SD* = 10.47) than Indian listeners (*M* = 9.05 taps, *SD* = 2.20) on trials with complex sequences, *F*(1,95) = 30.36, *p*<.001, *η_p_2* = .24. This suggests that even while listeners may eventually reach the same level of relative phase in culturally familiar and unfamiliar meters, they take significantly longer to do so when the pattern is based on an unfamiliar meter.

### Switch trials

#### Relative Phase

**Figure 7:**
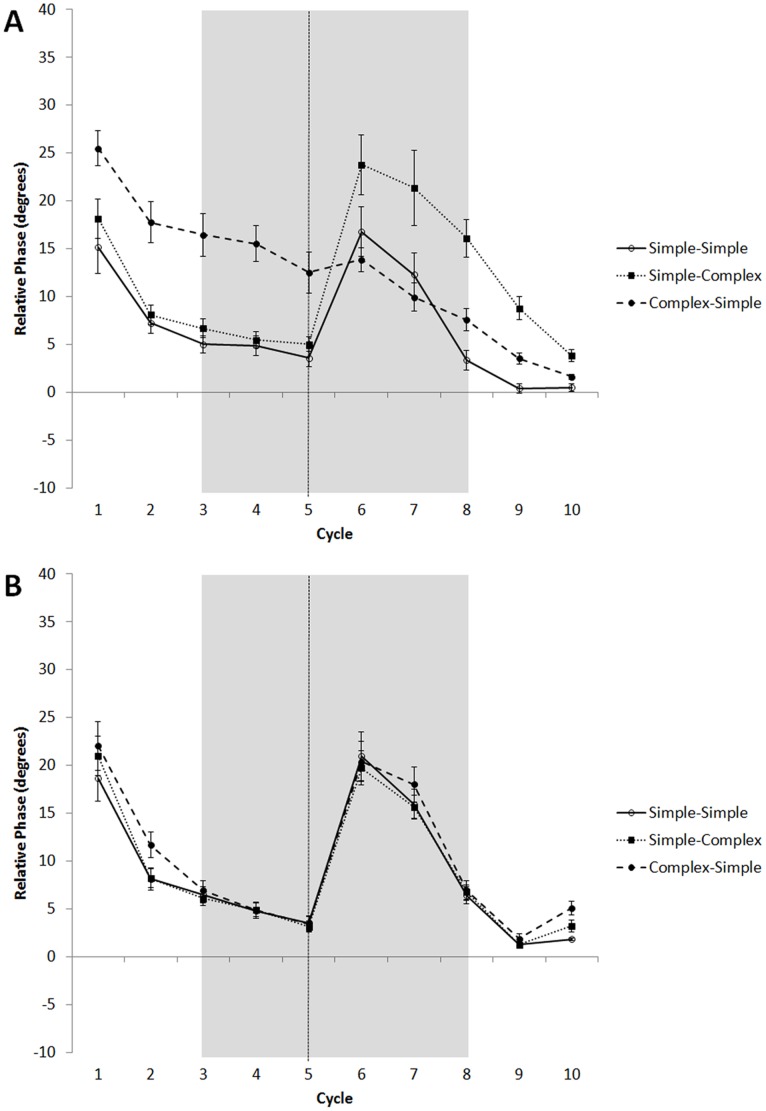
Mean average relative phase over course of trial for filled switch sequences for Americans (A) and Indians (B) in Experiment 1: simple-simple, simple-complex, and complex-simple conditions. Error bars denote between-subject standard error. Some error bars were too small to be visible. Dashed line denotes point of switch. Shaded box denotes window of interest in analyses.

Relative phase values for the six switch sequences were collapsed into three conditions: 1) simple-simple (averaged across duple-triple and triple-duple), 2) simple-complex (averaged across duple-complex and triple-complex), and 3) complex-simple (averaged across complex-duple and complex-triple). Figure 7 shows that the first half of switch trials was similar to baseline trials, with positive asynchronies generally decreasing with each successive tap. When the accompanying sequence suddenly changed after the 5^th^ target, asynchronies rose sharply depending on the condition and nationality of the participants.

**Figure 8:**
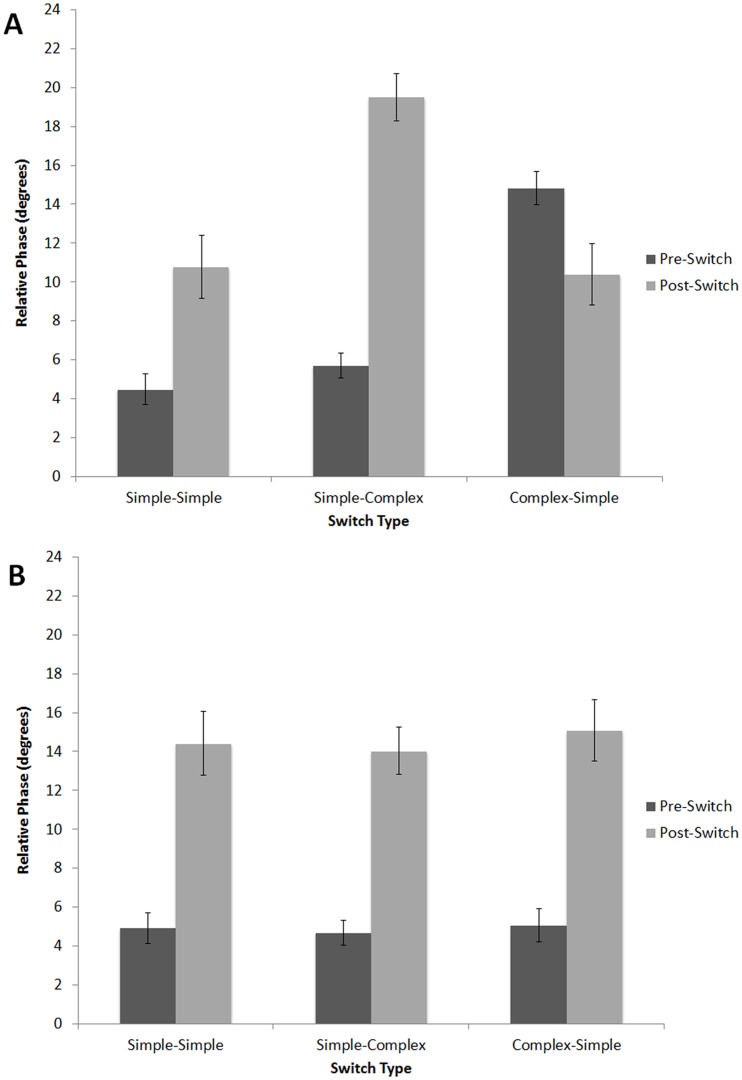
Mean relative phase (degrees) before and after the switch for Americans (A) and Indians (B) in Experiment 1: simple-simple, simple-complex, and complex-simple conditions. Error bars denote between-subject standard error.

To measure the effects of switching the sequence type halfway through the trial, we compared the mean relative phase over the three taps before and the three taps following the switch (see Figure 8). A 3×2×2 (Switch Condition [complex-simple, simple-simple, simple-complex], within-subjects, x Position [before switch, after switch], within-subjects, x Nationality [Indian, American], between subjects) mixed-design ANOVA, with Music Training as a covariate, revealed significant main effects of Position, *F*(1,99) = 146.57, *p*<.001, *η_p_2* = .59, Switch Condition, *F*(2,98) = 16.74, *p*<.001, *η_p_2* = .15, and Music Training, *F*(1,99) = 18.19, *p*<.001, *η_p_2* = .15. These effects were driven by relative phase values that were lower before (*M* = 6.55°, *SD* = 8.77) than after the switch (*M* = 13.97°, *SD* = 19.98), and by lower relative phase values in the simple-simple condition (*M* = 8.53°, *SD* = 9.09) than in the simple-complex (*M* = 10.90°, *SD* = 23.01) or complex-simple (*M* = 11.34°, *SD* = 11.31) conditions. Participants with more music training had lower relative phase values across all conditions, *r*s(102)<−.33, *p*s<.001.

There was a significant three-way interaction between Switch Type, Position, and Nationality, *F*(2,98) = 48.37, *p*<.001, *η_p_2* = .33. To explore this interaction, subsequent 3×2 (Switch Condition [complex-simple, simple-simple, simple-complex], within-subjects, x Position [before switch, after switch], within-subjects) ANOVAs, with Music Training as a covariate, were performed separately for each group. For American listeners, there were significant main effects of Position, *F*(1,49) = 43.37, *p*<.001, *η_p_2* = .47, Switch Type, *F*(1,49) = 30.11, *p*<.001, *η_p_2* = .38, and Music Training, *F*(1,49) = 18.63, *p*<.001, *η_p_2* = .28, as well as a significant interaction between Position and Switch Condition, *F*(2,48) = 44.36, *p*<.001, *η_p_2* = .48. Post-hoc two-tailed *t*-tests confirmed that the Position x Switch Condition interaction was driven by a significant *increase* in relative phase following the switch in the simple-simple, *t*(50) = 6.36, *p*<.001, and simple-complex conditions, *t*(50) = 7.92, *p*<.001, but a significant *decrease* in relative phase following the switch in the complex-simple condition, *t*(50) = 4.97, *p*<.001, as observed in Figure 8A. Indian listeners exhibited significant main effects of Position, *F*(1,49) = 105.48, *p*<.001, *η_p_2* = .68, and Music Training, *F*(1,49) = 6.69, *p*<.05, *η_p_2* = .12, but no significant interaction between Switch Type and Position, *F*(2,48) = 2.85, *p* = .063, *η_p_2* = .055. That is, unlike American participants, Indian listeners showed an increase in relative phase following the switch regardless of the type of switch (simple-simple, *t*(51) = 10.39, *p*<.001, simple-complex, *t*(51) = 15.00, *p*<.001, and complex-simple, *t*(51) = 11.33, *p*<.001, as observed in Figure 8B.

#### Tapping Variability

**Figure 9:**
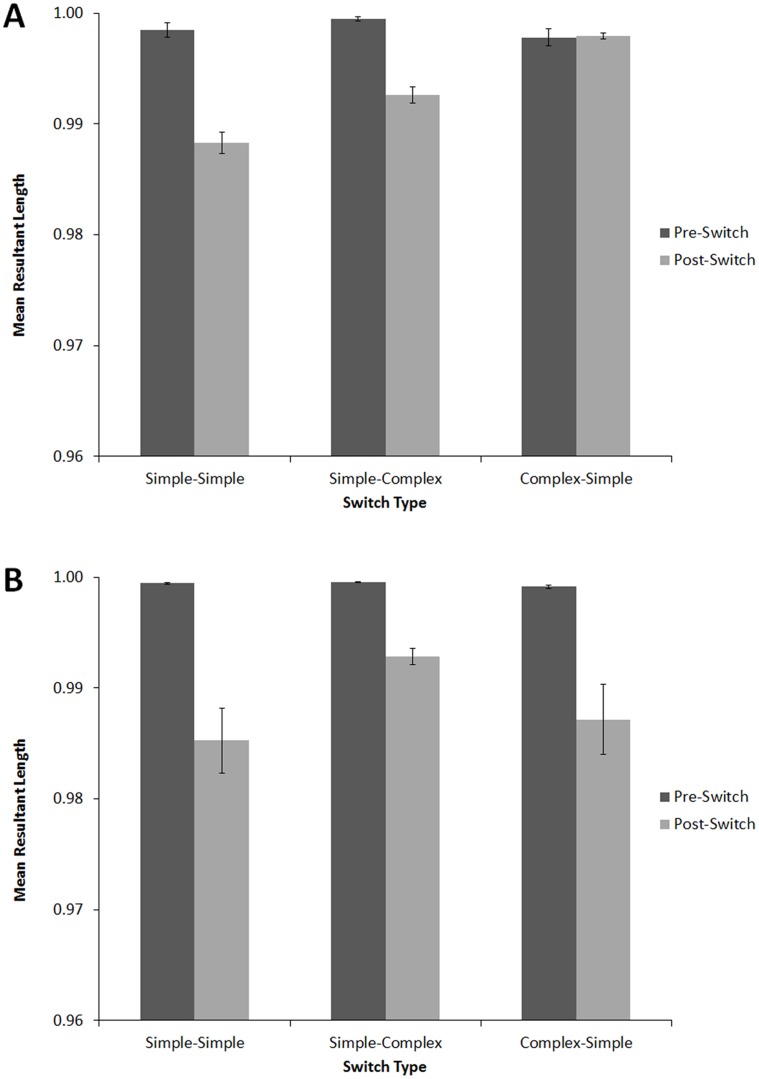
Angular variability (MRL) before and after the switch for Americans (A) and Indians (B) in Experiment 1: simple-simple, simple-complex, and complex-simple conditions. Error bars denote between-subject standard error.

MRL values were submitted to a 3×2×2 (Switch Condition [complex-simple, simple-simple, simple-complex], within-subjects, x Position [before switch, after switch], within-subjects, x Nationality [Indian, American], between subjects) mixed design ANOVA, with Music Training as a covariate (see Figure 9). There were significant main effects of Position, *F*(1,99) = 9.26, *p*<.001, *η_p_2* = .086, and Nationality, *F*(1,99) = 10.95, *p*<.01, *η_p_2* = .10. MRL values were higher before (*M* = .999, *SD* = .016) than after the switch (*M* = .991, *SD* = .020), indicating that tapping became more variable after the switch. For between-subjects effects, MRL values were slightly higher for American (*M* = .996, *SD* = .013) than for Indian participants (*M* = .994, *SD* = .022).

There was a significant three-way interaction between Switch Type, Position, and Nationality, *F*(2,98) = 5.86, *p*<.05, *η_p_2* = .056. We therefore performed separate 3×2 (Switch Condition [complex-simple, simple-simple, simple-complex] x Position [before switch, after switch]) ANOVAs for each Nationality, keeping Music Training as a covariate. For American listeners, there was a significant main effect of Switch Type, *F*(1,49) = 34.86, *p*<.001, *η_p_2* = .33, Position, *F*(1,49) = 84.10, *p*<.001, *η_p_2* = .64, and a significant interaction between Position and Switch Condition, *F*(2,48) = 24.74, *p*<.05, *η_p_2* = .34. Post-hoc two-tailed Bonferroni-corrected t-tests confirmed that the Position x Switch Condition interaction was driven by a significant decrease in MRL following the switch in the simple-simple, *t*(50) = 9.41, *p*<.001, and simple-complex conditions, *t*(50) = 9.97, *p*<.001, but no change following the switch in the complex-simple condition, *t*(50) = .12, *p* = .91. Indian listeners also exhibited a main effect of Position, *F*(1,49) = 67.00, *p*<.001, *η_p_2* = .58, but unlike American listeners, they showed no main effect of Switch Condition, *F*(2,48) = 2.88, *p* = .07, *η_p_2* = .056, and the interaction between Position and Switch Condition was not significant, *F*(2,48) = 2.64, *p* = .076, *η_p_2* = .051. Indian listeners exhibited higher variability following the switch regardless of the nature of switch (simple-simple, *t*(50) = 4.83, *p*<.001, simple-complex, *t*(50) = 9.41, *p*<.001, and complex-simple, *t*(50) = 3.76, *p*<.001).

## Discussion

Our results are consistent with the conclusion that listeners have an easier time tapping to a very slow 3-s tempo when intervals are filled with rhythmic sequences than when intervals are filled with silence. In the unfilled trials, participants showed a significant anticipatory tendency, as evidenced by large negative asynchronies between the tones and the participants' taps. In contrast, the presence of subdivisions clearly improved performance (as shown by lower relative phase). While it is possible that listeners are simply better at synchronizing to filled than unfilled intervals, the nature of subdivisions was key, and interacted with participants' prior experience with similar subdivision patterns. Thus, whereas Indian participants readily synchronized to simple- *and* complex-meter trials, Americans, whose experience was almost exclusively limited to simple meters, were better at and faster to synchronize during simple- than complex-meter trials. It is nonetheless noteworthy that American listeners *did* show a decline in relative phase (i.e., evidence of stabilization) during unfamiliar complex-meter trials, even though stabilization took longer for complex than for simple meter trials.

The switch trials provide even stronger evidence for the role of cultural experience. Although the presence of culturally familiar metrical structure clearly promotes greater synchrony and more rapid stabilization of synchronization, we predicted that the robustness of the inferred metrical framework would also contribute to the disruptive effects of an abrupt change of rhythmic pattern. After an abrupt switch away from a robustly activated meter, the new rhythm should seem syncopated, and thus disrupt synchronization while increasing variability [28]. By contrast, if the first rhythm does not (strongly) activate a metrical framework, there is presumably minimal syncopation following a switch and thus minimal disruption to synchronization. Consistent with these predictions, we found that an abrupt switch away from a simple meter disrupted synchronization among all listeners, as demonstrated by increases in relative phase and variability after the switch. On the other hand, following a switch away from a complex meter, only Indian listeners showed a disruption in performance. This suggests that Americans were unable to activate a robust metrical framework for the complex-meter rhythms. Musicianship also highlights the role of experience in synchronizing with these sequences, with music training negatively correlated with variability and relaxation time, but only for the complex sequence condition. That is, musicians are less variable and faster to synchronize with these complex sequences.

What remains unclear is how experienced Western listeners process rhythms conforming to complex meters, and whether or not they can ever infer a complex meter. A prior study provided adult American listeners with at-home exposure to complex-meter music for two weeks, and their ability to perceive subtle disruptions to complex- and simple-meter melodies was compared before and after exposure [23]. Adult listeners exhibited minimal change in discrimination after this exposure, whereas younger American listeners (age 12 months to 6 years) showed significant improvement [23], [46]. This implies that perhaps there is a sensitive or optimal period of learning that applies to acquisition of knowledge about temporal structure in music, as appears for acquisition of some components of a second language [47]. Alternatively, all listeners may have the capacity to process both simple and complex meters, even if they exhibit a bias towards familiar meters. This possibility is supported by the present findings in two ways. First, we found that relative to unfilled intervals, complex-meter baseline trials yielded smaller, more positive asynchronies among both Indian and American participants, suggesting all listeners benefitted from the presence of the complex-meter rhythm. Second, although relative phase was elevated throughout baselines trials for complex- as opposed to simple-meter sequences, it nevertheless declined dramatically by the end of the 10 cycles. That is, Americans *did* eventually stabilize their synchrony during complex-meter trials, even though they did so at a slower rate than for simple-meter trials. If American participants were given more time for their synchronization tapping to stabilize, they too might show a cost of switching away from complex meter. To address this question, we used the same basic paradigm from Experiment 1 with a new set of American participants, but this time we gave them more time to stabilize their synchronization tapping prior to the switch.

### Experiment 2

In this experiment, we doubled the pre- and post-switch durations in each trial to determine whether a cost of switching would be evident if participants were able to attain greater stability of synchronization prior to the switch. If the lack of culture-specific exposure to complex meters prevents American listeners from being able to infer any helpful representation of the complex-meter sequences, then Americans in this experiment should exhibit no cost of switching from complex meter even after ten cycles of tapping to complex-meter patterns. On the other hand, if American listeners have the capacity to process and benefit from complex-meter sequences despite their culture-specific biases towards simple meters, they should show a cost of switching away from unfamiliar complex meters given sufficient time for pre-switch synchronization tapping to stabilize.

#### Participants

Participants were 17 college students from Las Vegas, Nevada, USA (M_age_ = 22.7 years, 8 male, 9 female) who participated for course credit. Their music training ranged from 0–8 years (*M* = 2.06, *SD* = 3.11), with 5 participants reporting 0 years of music training.

#### Apparatus and Stimuli

The stimuli were created similarly to Experiment 1, except that instead of 5 cycles, there were 10 cycles before and 10 cycles after the switch.

#### Procedure

The procedure was identical to that of Experiment 1 except that only the six switch sequences were presented. No silent or filled baseline trials were presented.

#### Data Analysis

As in Experiment 1, the last 3 taps prior to the switch, and the first 3 taps following the switch were used in calculating the mean relative phase values and MRL. All other analyses were identical to that of Experiment 1. A total of 0.73% of taps were excluded for being more than five standard deviations away from the mean relative phase. Because there was only one cultural group and because we had so few musicians (only 5 participants with 5 or more years of formal training) we did not include music training as a covariate in any analyses, however we computed correlations between music training and each dependent measure for each trial type.

## Results

### Relaxation time

**Figure 10:**
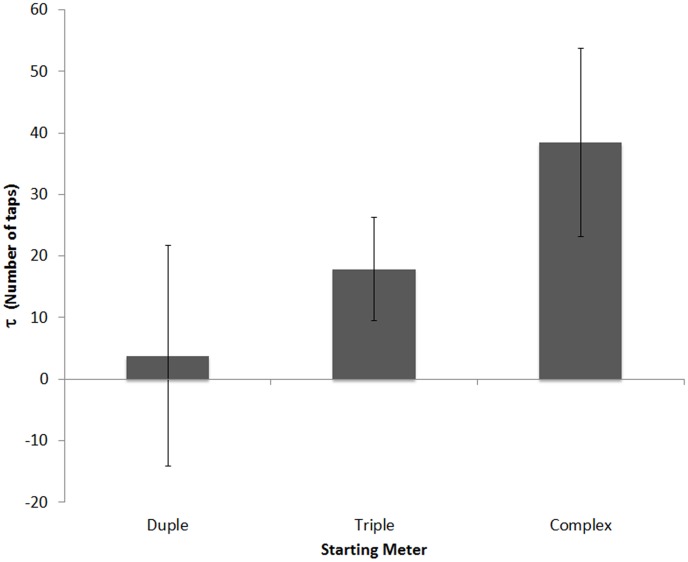
Relaxation time: τ (in number of taps) for sequences starting with duple, triple and complex sequences in Experiment 2 (Americans only). Values represent an estimate of the number of cycles (taps) required for relative phase to reach 1 degree. Error bars denote within-subject standard error [51].

As in Experiment 1, relaxation time was measured using all pre-switch tap times, averaged based on starting sequence, to yield a separate τ for starting duple, starting triple, and starting complex conditions. Resulting τ values that were more than 5 standard deviations away from the overall mean were excluded as outliers (2.71% of all τ values). A one-way (Starting Sequence [duple, triple, complex]) repeated-measures ANOVA on τ values revealed a significant effect of Starting Sequence, *F*(2,15) = 3.734, *p*<.05, *η_p_2* = .189. Paired-sample t-tests revealed differences between complex (M = 38.47, SD = 28.50) and triple (M = 17.91, SD = 27.99) sequences, *t*(16) = 2.37, *p*<.05, marginal differences between complex and duple (M = 3.75, SD = 42.59) sequences, *t*(16) = 2.11, *p* = .051, but no differences between duple and triple sequence conditions, *t*(16) = 1.18, *p* = .25. Consistent with Experiment 1, relaxation time was greatest for complex-meter trials (see Figure 10). There were no significant correlations between music training and τ values for any trial type.

### Relative Phase

**Figure 11:**
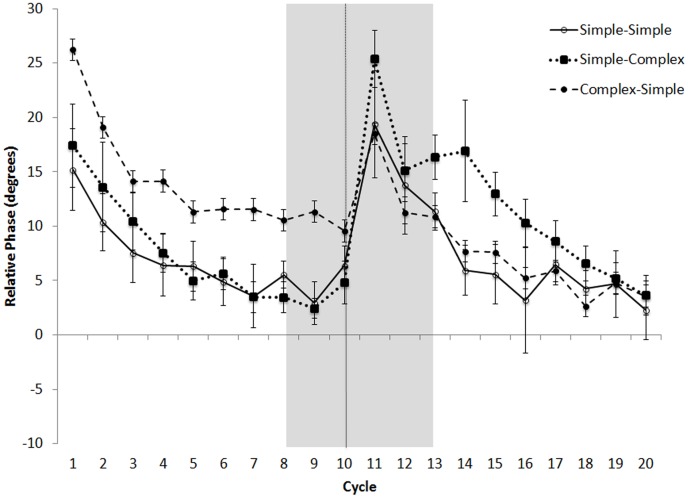
Mean relative phase over course of trial for filled switch sequences in Experiment 2 (Americans only): simple-simple, simple-complex, and complex-simple conditions. Error bars denote within-subject standard error.

Relative phase for the six filled switch sequences were collapsed into three conditions, as in Experiment 1: 1) simple-simple, 2) simple-complex, and 3) complex-simple. Figure 11 depicts mean relative phase in the above three conditions. Similar to the results from Experiment 1 and consistent with the relaxation time analysis reported above, relative phase values were lower and declined more rapidly when the first half of the trial contained a simple than a complex pattern. Following the switch, there was an increase in relative phase following a switch from the simple meter, but also an increase in relative phase following the switch from the complex meter.

**Figure 12:**
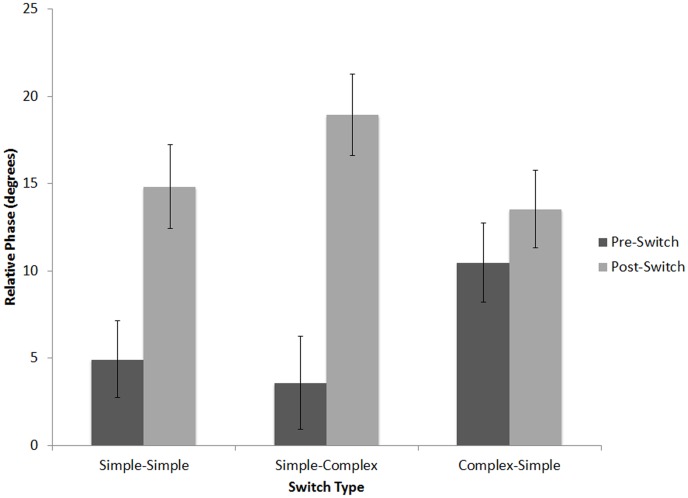
Mean relative phase before and after the switch in Experiment 2 (Americans only): simple-simple, simple-complex, and complex-simple conditions. Error bars denote within-subject standard error.

Using mean relative phase of the 3 taps immediately preceding and the 3 taps immediately following the switch, we conducted a 3×2 (Switch Condition [complex-simple, simple-simple, simple-complex], x Position [before switch, after switch]) repeated-measures ANOVA, which revealed a significant main effect of Position, *F*(1,15) = 158.89, *p*<.001, *η_p_2* = .909, with larger relative phase values after the switch (*M* = 15.76°, *SD* = 6.00) than before (*M* = 6.33°, *SD* = 6.31). There was also a significant interaction between Switch Type and Position, *F*(2,15) = 28.497, *p*<.001, *η_p_2* = .64. Although relative phase increased in all three conditions after the switch, whether complex-simple, *t*(16) = 2.15, *p*<.05, simple-simple, *t*(16) = 8.86, *p*<.001, or simple-complex, *t*(50) = 14.81, *p*<.001, Figure 12 indicates that the disruptive effects of the switch varied across conditions. Difference scores were calculated by subtracting pre-switch from post-switch relative phase for each condition. A one-way (Switch Condition [complex-simple, simple-simple, simple-complex]) repeated-measures ANOVA was significant, *F*(2,15) = 28.497, *p*<.001, *η_p_2* = .64. Difference scores were larger when switching from simple to complex (*M* = 15.37°, SD = 4.38) than when switching from simple to simple (*M* = 9.88°, SD = 4.6), *t*(16) = 3.951, *p*<.001. Likewise, switching from complex to simple yielded a smaller difference score (*M* = 3.06°, SD = 5.87) than for either of the other two conditions, *t*s(16)>3.77, *p*<.01. Thus, despite a cost of switching in all three conditions, the cost was larger when the trial began with simple than when it began with complex-meter patterns. There were again no significant correlations between music training and relative phase for any trial type.

### Tapping Variability

**Figure 13:**
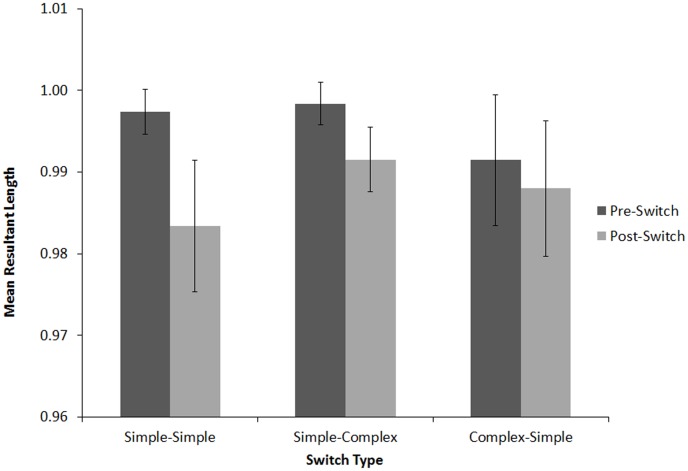
Angular variability (MRL) before and after the switch in Experiment 2 (Americans only): simple-simple, simple-complex, and complex-simple conditions. Error bars denote within-subject standard error.

As in Experiment 1, the last 3 taps prior to the switch were used to calculate the pre-switch MRL values, and the first 3 taps following the switch were used to calculate the post-switch MRL values (Figure 13). Similar to Experiment 1, MRL values were close to 1, indicating generally low variability across all conditions. MRL values were submitted to a 3×2 (Switch Condition [complex-simple, simple-simple, simple-complex], x Position [before switch, after switch]) repeated-measures ANOVA. There was a significant main effect of Position, *F*(1,16) = 9.503, *p*<.01, *η_p_2* = .373, with a significantly lower MRL value after the switch (*M* = .988°, *SD* = .016) than before (*M* = .996°, *SD* = .011), indicating that tapping became more variable after the switch, in line with findings from Experiment 1 (see Figure 13). There were no other significant main effects or interactions.

Only in the complex-simple condition after the switch was there a correlation between music training and variability, *r*(17) = −.492, *p*<.05, indicating that tapping by individuals with more musical training was more variable after a switch from complex to simple meter.

## Discussion

In Experiment 1, we showed that Indian listeners exhibited a cost of switching (i.e., increase in relative phase) following any switch, whereas American listeners showed an increase in relative phase only after a switch away from a simple meter, and no cost was associated with switching away from a complex meter. We interpreted this to suggest stronger representations in Americans for simple than for complex meters. However, even in the complex meter trials, Americans showed a general tendency for the relative phase values to approach 0, albeit more gradually, as evidenced by the relaxation time measurements. Thus, an open question was whether longer periods of familiarization would enable Americans to reach a comparable level of relative phase on the simple and complex trials, and if they still would show a similar cost of switching away from simple and complex meters. Experiment 2 therefore asked American participants to synchronize with 10 cycles (as opposed to 5 cycles as in Experiment 1) before a switch in the pattern.

In this experiment, Americans again showed a decrease in relative phase during the first half of complex-meter trials that approached (but did not reach) zero, and relaxation time was longer for the complex than simple meter trials, consistent with Experiment 1 (see Figure 10). Unlike Experiment 1, there was a cost of switching meters for all switch types (see Figure 11), although the cost was larger for the simple to complex switch type. Taken together, these results suggest that a lack of experience with complex meters makes it more difficult, but not impossible to benefit from the presence of a complex-meter sequence. It is possible that even without exposure to complex-meter music in one's culture, listeners possess a basic capacity to form some type of cognitive representation based on predictable features of a sequence that, with repeated exposure, allows them to learn from and predict when targets will occur in time with increasing precision as the trial progresses. This representation, once internalized, can be disrupted by an abrupt switch in meter type that has qualitatively similar (though quantitatively different) effects on performance across simple and complex meter trials, even for listeners unfamiliar with the complex meters.

## General Discussion

The present experiments shed light on how culture-specific experience and knowledge influence a fundamental musical behavior—synchronous tapping to a rhythmic stimulus. We employed a novel task in which we instructed participants to tap synchronously with target tones and ignore the context surrounding target tones, yet we found that to-be-ignored rhythmic sequences powerfully influenced synchronization. To some extent, this could be due to the filled-duration illusion, where filled intervals are perceived to be longer than unfilled intervals, and are therefore shortened in production tasks [48]. However, the effect was clearly driven by familiarity with the metrical pattern, as observed by the differential performance on simple and complex meters by Indians and Americans. This suggests that listeners found it difficult not to integrate the to-be-ignored rhythmic pattern into the task.

Our experiments allowed us to address unresolved questions about the cognitive difficulty posed by non-isochronous, complex musical meters compared with isochronous, simple meters [14]–[21]. Although this bias is well documented, it remains unclear whether limitations arise from inexperience with complex meters [23], biases intrinsic to listeners or to the stimuli themselves [14], [25]–[26], or some combination of the two. Our findings suggest that the cultural familiarity of a distractor sequence's implied meter both facilitated and interfered with synchronization performance (Experiment 1), but we also provide evidence that Western listeners can nevertheless benefit from short-term exposure within a trial to a predictable but unfamiliar sequence (Experiment 2).

In the first experiment we showed that presenting participants with to-be-ignored rhythmic sequences alters how they synchronize their taps to a target that occurs once every 3 seconds. On average, taps occurred early (negative asynchronies) when the inter-target interval was silent (Figure 4). By contrast, taps occurred late (positive asynchronies) during filled baseline sequences, with culturally familiar sequences generating much smaller asynchronies than culturally unfamiliar sequences. Asynchronies declined over the course of baseline trials; however, as evidenced by the relaxation time measure, this decline was much more gradual for Americans in the complex-meter condition than in the simple-meter conditions. That is, it took longer for synchronization performance to stabilize when the meter of the sequence was culturally foreign, suggesting that the representation for meter was less stable in the unfamiliar, complex meter context. By contrast, participants from India performed virtually the same way across all filled baseline conditions, presumably because both complex- and simple-meter conditions were culturally familiar, suggesting that their representations for both simple, and complex meters were equally stable.

The filled switch trials allowed us to examine the effects of an abrupt switch in the metrical pattern halfway through the trial, which we expected would sound syncopated if listeners had activated a robust representation of the meter presented during the first half of the trial. These switch trials allowed us to more directly measure the degree of stability of these meters. We thus predicted that the highest cost of switching would be seen when the first half of the trial contained a culturally familiar meter. This is indeed what we found: when the trial began with a simple meter that was culturally familiar to both groups (triple, for example) and then abruptly shifted to a contrasting simple, familiar meter (duple, for example), Indian and American listeners both exhibited a cost of switching, which was manifested by increased relative phase (Figure 8) as well as increased variability (Figure 9), pointing to a large degree of stability for the pre-switch simple meter. Group differences only emerged when a switch trial contained a complex-meter sequence. When the complex-meter sequence occurred during the second half of the trial, Americans' cost of switching was larger than with a switch from duple to triple (or vice versa), and the cost was also larger for Indian listeners in the same condition. When the complex-meter sequence was present during the first half of the trial, only Indian listeners exhibited a cost of switching, whereas American listeners actually showed lower asynchronies after switch and their tapping variability was unaffected by the switch.

An ambiguity in the above findings is that for the complex-simple switch trials, American participants had encountered the switch before their synchronization performance had an opportunity to stabilize. Although differential patterns of stabilization over time for simple- versus complex-meter sequences strongly implicate a key role of culture-specific metrical representations in synchronization, it is nevertheless possible that the observed cost of switching had more to do with the stability of synchronization at the time of the switch than the disruption of culture-specific metrical representations per se. It might be that any type of rhythmic predictability in a sequence will lead to stabilized synchronization performance if enough time is given. The second experiment therefore directly addressed this ambiguity by doubling the length of the trial and presenting the switch after ten cycles. Experiment 2 revealed that Americans did exhibit a cost of switching even when switching away from a complex-meter sequence, suggesting that despite the lack of cultural familiarity of the starting pattern, they still benefitted from the predictability of the foreign sequence and thus experienced destabilization when they abruptly heard a new, culturally familiar sequence. That is, when given enough time to familiarize with an unfamiliar but predictable sequence, listeners were able to stabilize a metrical representation.

It was also clear from Experiment 2, however, that even though all switch conditions produced a cost of switching, the cultural familiarity of the sequence influenced the size of the cost of switching. The smallest cost was observed when switching from complex to simple, which could have risen in part from the fact that synchronization was still less complete—even after 10 cycles—for trials starting with complex than for those starting with simple sequences. However, this does not explain why, in Experiment 1, the cost of switching from simple to complex was larger than the cost of switching from simple to simple for American listeners but not Indian listeners. The latter result strongly implies that the cultural familiarity of the post-switch meter also influences how quickly an increase in relative phase can recover from a disruption. Nevertheless, to the extent that observed post-switch increases in relative phase and variability can be attributed to syncopation or reorganization of a metrical percept, culturally unfamiliar meters can also give rise to a metrical percept and syncopation if enough exposure is provided. This result provides novel evidence that despite the powerful role of culture-specific experience and knowledge, even the minimal exposure to complex meters within the course of the experiment can change the way listeners respond to unfamiliar but predictable rhythms. This points to the role of learning, whether via the life-long culture-specific learning seen for familiar meters, or the learning that occurs within the course of an experiment.

One remaining concern is that some of the findings might be attributed to group differences unrelated to meter and culture. For example, the Indian participants had slightly more (∼1 year) musical training than did American participants on average, which might have led them to be more proficient at synchronization in all conditions. We therefore included music training as a covariate in all Experiment 1 analyses but nevertheless observed group differences in conditions involving complex meter. It is also possible that because Indian music can contain extremely slow tempi and long metrical cycles [20], Indian listeners might have had greater experience with long-interval timing. However, both groups performed poorly on unfilled trials, and both groups benefited from the presence of intervening rhythmic patterns that conformed to familiar meters (triple and duple). Thus, performance by both groups on unfilled and filled simple meter baseline sequences suggests that neither musical training nor exposure to slow tempi was sufficient to override basic processing limits based on tempo [31], [32]. Despite our use of a sensitive, on-line measure of performance than those used in previous studies [23], we found no evidence that Indian listeners perceive or represent complex meters differently than they do simple meters. Indian listeners exhibited advantages of activating simple and complex meters, and costs associated with being forced to abruptly reorganize those metrical percepts. Thus, this study is consistent with other recent evidence [25] that the processing of simple and complex meters is comparable when culturally familiar.

In conclusion, our findings reveal a more nuanced depiction of the difficulty with which Western listeners perceive and produce non-isochronous complex meters [23], [49], [50]. We suggest that this difficulty arises in part from intrinsic biases towards simple, isochronous meters, but also from learning processes that take place over the course of child's development within a particular culture (Experiment 1) or over the course of a trial or experiment (Experiment 2). Presumably, complex meters are harder to represent when they are unfamiliar to listeners, and perhaps harder to learn, which makes American listeners' synchronization behavior less stable. If simple meters are intrinsically easier to learn in comparison with complex meters, this might account for the widespread use of simple meters in every studied culture, including cultures that have a large presence of complex meters in their music. While several cultures exclusively use simple meter ratios in their music, no known musical culture uses exclusively complex meter ratios. Our findings provide compelling evidence that despite any possible intrinsic biases for simple meters, both simple and complex meters can be represented similarly and can be inferred with equal strength by listeners for whom they are familiar, consistent with previous research using perceptual judgments [26].
